# Adaptive vs. Conventional Deep Brain Stimulation: One-Year Subthalamic Recordings and Clinical Monitoring in a Patient with Parkinson’s Disease

**DOI:** 10.3390/bioengineering11100990

**Published:** 2024-09-30

**Authors:** Laura Caffi, Luigi M. Romito, Chiara Palmisano, Vanessa Aloia, Mattia Arlotti, Lorenzo Rossi, Sara Marceglia, Alberto Priori, Roberto Eleopra, Vincenzo Levi, Alberto Mazzoni, Ioannis U. Isaias

**Affiliations:** 1 Parkinson Institute of Milan, ASST G.Pini-CTO, 20126 Milano, Italy; 2 University Hospital of Würzburg and Julius Maximilian University of Würzburg, 97070 Würzburg, Germany; 3 The BioRobotics Institute, Sant’Anna School of Advanced Studies, 56025 Pisa, Italy; 4 Parkinson and Movement Disorders Unit, Foundation IRCCS Istituto Neurologico Carlo Besta, 20133 Milano, Italy; 5 Newronika S.p.A., 20093 Milano, Italy; 6 Department of Engineering and Architecture, University of Trieste, 34127 Trieste, Italy; 7 Department of Health Sciences, Aldo Ravelli Research Center for Neurotechnology and Experimental Neurotherapeutics, University of Milan, 20122 Milano, Italy; 8 Functional Neurosurgery Unit, Foundation IRCCS Istituto Neurologico Carlo Besta, 20133 Milano, Italy; 9 Department of Excellence in Robotics and AI, Sant’Anna School of Advanced Studies, 56127 Pisa, Italy

**Keywords:** parkinson’s disease, adaptive deep brain stimulation, neuromodulation, subthalamic nucleus, local field potentials, biomarkers

## Abstract

Conventional DBS (cDBS) for Parkinson’s disease uses constant, predefined stimulation parameters, while the currently available adaptive DBS (aDBS) provides the possibility of adjusting current amplitude with respect to subthalamic activity in the beta band (13–30 Hz). This preliminary study on one patient aims to describe how these two stimulation modes affect basal ganglia dynamics and, thus, behavior in the long term. We collected clinical data (UPDRS-III and -IV) and subthalamic recordings of one patient with Parkinson’s disease treated for one year with aDBS, alternated with short intervals of cDBS. Moreover, after nine months, the patient discontinued all dopaminergic drugs while keeping aDBS. Clinical benefits of aDBS were superior to those of cDBS, both with and without medications. This improvement was paralleled by larger daily fluctuations of subthalamic beta activity. Moreover, with aDBS, subthalamic beta activity decreased during asleep with respect to awake hours, while it remained stable in cDBS. These preliminary data suggest that aDBS might be more effective than cDBS in preserving the functional role of daily beta fluctuations, thus leading to superior clinical benefit. Our results open new perspectives for a restorative brain network effect of aDBS as a more physiological, bidirectional, brain–computer interface.

## 1. Introduction

Deep brain stimulation (DBS) of the subthalamic nucleus (STN) is a mainstay non-pharmacological treatment for selected Parkinson’s disease (PD) patients [1,2]. Currently, the DBS paradigm is conventional DBS (cDBS), which is based on uninterrupted stimulation with clinically determined fixed electrical settings (i.e., amplitude, pulse width, frequency, and wave form), unrelated to the continuously changing functional state of the brain. Such cDBS programming aims to improve the main motor Parkinsonian symptoms; however, the need to avoid unwanted, stimulation-related adverse effects may result in suboptimal clinical responses in some cases [3,4,5,6]. Adaptive DBS (aDBS) has the potential to optimize stimulation delivery through a responsive neuromodulation strategy, i.e., adapting stimulation parameters in a real-time manner by acquiring and elaborating symptom-specific and task-related biomarkers [2,7]. To date, the most promising brain biomarkers in PD patients are the local field potentials (LFPs) recorded directly from implanted DBS electrodes. Strong oscillatory beta activity (13–30 Hz) of STN-LFPs could be a valid biomarker for bradykinesia and rigidity, as it is associated with the severity of PD-related motor symptoms [8] and symptom improvement due to levodopa administration [9] and STN-DBS [10].

Preliminary clinical evidence in short time windows suggests superior clinical efficacy of aDBS over cDBS in treating PD-related motor symptoms [11,12,13,14,15,16] with fewer stimulation side effects compared to cDBS [17]. However, data on the long-term efficacy and safety of aDBS are still lacking, and its mechanism of action is still poorly understood. Accordingly, we describe the clinical and neurophysiological data collected over 11 months of follow-up in a patient with PD who underwent implantation of the AlphaDBS device (Newronika S.p.A.). Throughout this period, we evaluated the different effects of the two types of stimulation (aDBS or cDBS) on motor signs, asleep or awake states, and dopaminergic medications and tried to decode the specificities of the recorded subthalamic beta activity.

## 2. Materials and Methods

### 2.1. Patient History

We report the case of a male patient with the onset of Parkinsonian signs (resting tremor and bradykinesia in the right hand) in his early 40s. Following the consistency of his clinical and symptomatologic evolution and the congruence of SPECT with FP-CIT imaging [18], the patient received a diagnosis of idiopathic PD and followed therapy with dopamine agonists, levodopa, and iMAO agents. However, the development of severe motor fluctuations with peak-dose dyskinesias necessitated bilateral STN-DBS seven years after the onset of symptoms. Quadripolar electrodes 3389 connected to an implantable pulse generator ([IPG], Activa SC 37603, Medtronic Inc., Minneapolis, MN, USA) were used with remarkable clinical benefit. After about four years, the patient started aDBS upon receiving the experimental AlphaDBS IPG (Newronika S.p.A., Milano, Italy) as a replacement for the original battery-depleted IPG. The study was approved by the Foundation IRCCS Istituto Neurologico Carlo Besta Local Ethics Committee and Milano Area 2 Ethics Committee (approval code: 165-2020 and 93-2023bis) and conformed to the declaration of Helsinki. The patient gave written informed consent prior to participation in the study and for publication of the data. The patient was treated in aDBS+ (i.e., dopaminergic treatment maintained and stable) for 11 months. During this period, sporadic switches from aDBS+ to cDBS+ were performed automatically by the device, following the detection of a false positive sensing failure. Unaware of having returned to cDBS+, the patient asked for a visit because of a reduction in the clinical benefit of stimulation; upon checking, aDBS+ was reactivated. After about 9 months of aDBS+ treatment, the patient requested to discontinue all dopaminergic medication (aDBS− condition), complaining of agitation and restlessness after taking them. Since then, the patient asked to maintain the discontinuation of drug therapy after judging the effect of aDBS− alone to be better than the aDBS+ condition.

### 2.2. Adaptive Paradigm and Programming of the AlphaDBS Device

In aDBS mode, the AlphaDBS device [19] applies a linear algorithm that changes the stimulation current every minute based on the average LFP amplitude calculated in a patient-specific beta frequency range and normalized for the total amplitude in the 5–34 Hz range. Specifically, the averaging procedure consists of an exponential moving average with a time constant of 50 s over the beta samples calculated with one-second resolution. The stimulation amplitude is adjusted within a predefined clinically effective range independent for the two STNs (i.e., Amin and Amax), while the stimulation frequency and pulse width remain fixed.

The left recording contact pair 0–2 was chosen in our patient as it showed the most prominent beta peak among all contact pairs. The frequency range monitored (11–16 Hz) was defined as ±2.5 Hz centered to the highest beta peak.

**Figure 1:**
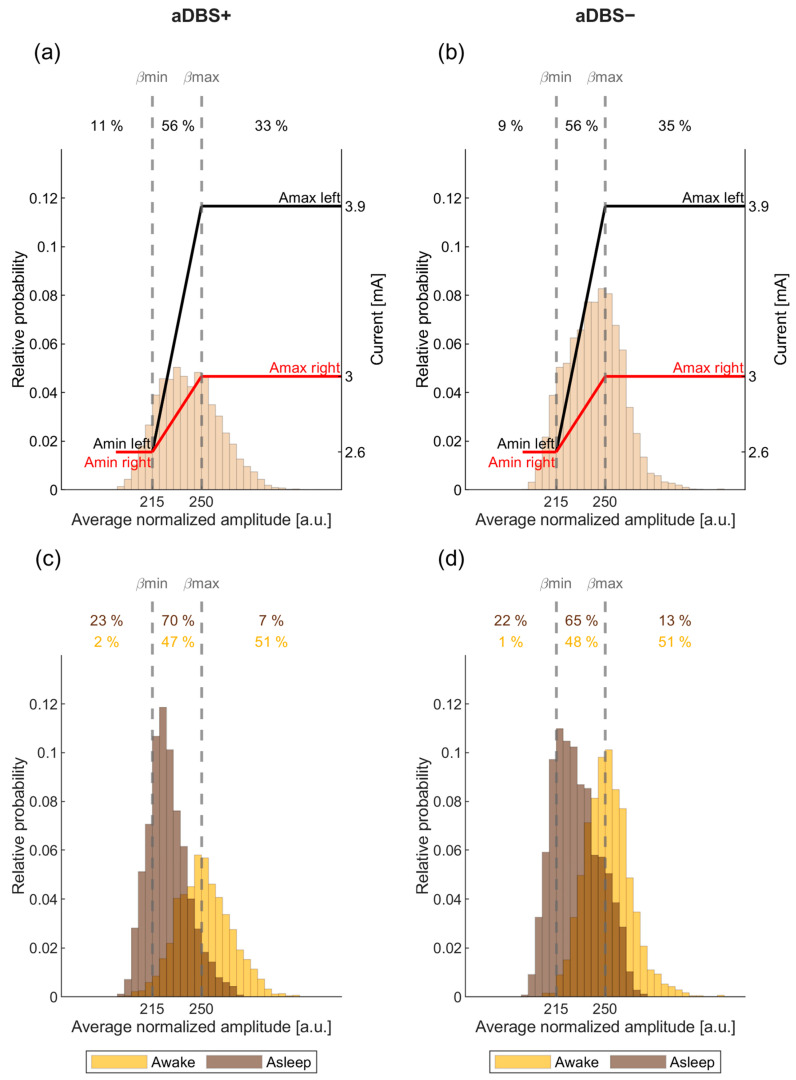
Principles of the AlphaDBS algorithm for current adjustment in adaptive mode. (**a**) Probability distribution of the biomarker (average normalized beta amplitude) used for current adjustment during a representative week in aDBS+ condition. Vertical dotted lines represent the biomarker limits for current adjustment (βmin and βmax). Red and black solid lines represent the stimulation current at a specific reading, respectively, for the right and left hemispheres. Current is adjusted within a predefined, clinically effective range (Amin–Amax). Numbers on top show the time percentage of the beta amplitude being less than βmin, between βmin and βmax, and above βmax in the considered week. (**b**) Same as (**a**) for the aDBS− condition. (**c**) Probability distribution of the biomarker during the same week in aDBS+ condition displayed in (**a**) separately for the awake (yellow) and asleep (brown) time. Time percentages are shown for both awake (yellow) and asleep (brown) time. (**d**) Same as (**c**) for the aDBS− condition. Abbreviations: a, adaptive; A, predefined, clinically effective amplitude; β, average normalized beta amplitude; c, conventional; DBS, deep brain stimulation; DBS+, with dopaminergic medication and DBS−, without dopaminergic medication.

The beta amplitude distribution in this frequency range was initially monitored for one day with cDBS+ and checked at different follow-up visits. This allowed the identification of the beta amplitude limits (βmin and βmax) by which the stimulation current was to be delivered in aDBS mode. In terms of programming options, these limits define the amount of time the patient is stimulated towards or at Amin or Amax (Figure 1). Specifically, the two beta amplitude limits were empirically defined to allow amplitude modulation with intermediate current values between Amin and Amax for approximately 50% of the total time.

The two stimulation current limits were clinically defined as the minimum amplitude (Amin) providing 40–50% clinical benefit in meds-off state (i.e., titrating up the stimulation current in the morning after overnight suspension of all dopaminergic drugs) and the maximum amplitude (Amax) in the absence of side effects in the meds-on condition (i.e., titrating up the stimulation current at 60 min after 100 + 25 mg levodopa + carbidopa intake).

Programming in cDBS was performed as a standard of care [20,21].

### 2.3. Spectral Analysis

With active stimulation, the AlphaDBS device saved the stimulation current and the average subthalamic amplitude spectrum (in the band 5–34 Hz with 1 Hz resolution) every ten minutes [19]. We analyzed the awake and asleep periods separately since a reduction of subthalamic beta power during sleep has been reported in patients treated with cDBS [22]. The same time windows for awake (8 a.m.–10 p.m.) and asleep (midnight to 6 a.m.), chosen according to the patient’s daily routine, were used for each day. Recordings from 6 a.m. to 8 a.m. and from 10 p.m. to midnight were excluded because of the differences in the patient’s daily schedule (e.g., time of falling asleep, etc.). To investigate relevant spectral peaks, we cleaned the amplitude spectra from 1⁄*fn* noise as follows. For each day, we computed two average spectra, one for awake and one for asleep time, by averaging the amplitude spectra acquired from 8 a.m. to 10 p.m. and from midnight to 6 a.m., respectively. The daily average spectra showed an aperiodic component superimposed on the oscillatory peaks. Consequently, after identification of the aperiodic component starting frequency (7 Hz) by visual inspection, the awake/asleep average spectra for each day were decomposed in the two components, aperiodic and periodic. These two components were modeled, respectively, as exponential functions with characteristic offset, slope, and bend, and Gaussian functions with characteristic central frequency, amplitude, and width [23]. The quality of the decomposition was visually inspected, and days presenting residual spectral artifacts after subtraction of the aperiodic component were removed by the analysis. Moreover, the presence and stability of the Gaussian peaks were inspected across days. For each day, spectral peaks were ranked according to frequency bands identified by visual inspection. In the case of multiple peaks belonging to the same frequency band, only the peak associated with the highest amplitude was kept. For further analysis, for each day, the daily awake or asleep periodic components were subtracted from each ten-minute amplitude spectrum acquired during the day. For each spectrum, we then calculated the mean amplitude in the patient-specific beta frequency range (BFRA).

### 2.4. Quantification and Statistical Analysis

Within each day, separately for the awake and asleep periods, we calculated the median and interquartile range of the BFRA of each ten-minute amplitude spectrum acquired during the day. The effects of treatment (cDBS+, aDBS+, and aDBS−) and patient’s status (awake and asleep) were evaluated using mixed ANOVA on the ranked data [24].

Subsequently, a single main effect analysis was conducted with non-parametric tests after checking for normality with the Anderson-Darling test. Differences between the treatment conditions, separately for awake and asleep periods, were assessed using the Kruskal-Wallis test. Post-hoc analysis was conducted using Tukey’s honestly significant difference procedure. The effect of the patient’s status (awake and asleep) in the different treatment conditions was conducted through the Wilcoxon signed-rank test. The statistical significance was set at 0.05. The effect size was calculated using Robust Cohen’s distance (rCoh), obtained from Cohen’s distance by replacing the population means with the 20% trimmed means and the population standard deviation with the square root of a 20% Winsorized variance [25]. Mutual information between the awake/asleep condition and BFRA features was computed based on standard methods using the Panzeri-Treves method for bias correction [26] and the bootstrap test for significance (significance if information > 95% percentile of the information data, 100 repetitions). All estimates were computed with the Information Breakdown Toolbox [27].

## 3. Results

### 3.1. Consistent and Sustained Long-Term Clinical Improvement with aDBS

**Figure 2:**
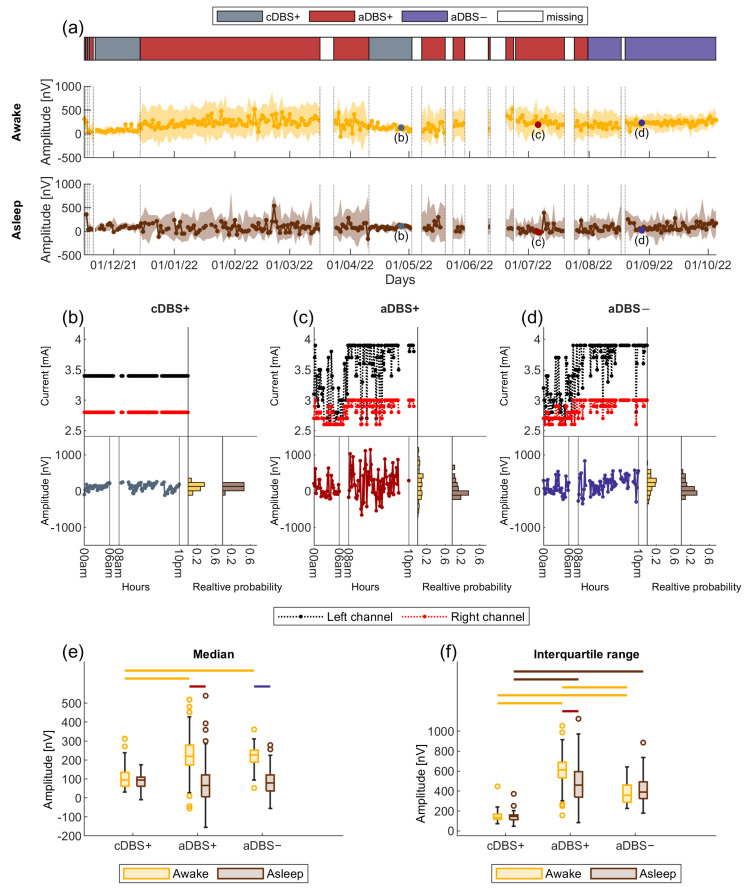
Beta amplitude modulations in aDBS than in cDBS. (**a**) Evolution of the daily median BFRA (solid line) during awake (yellow) and asleep (brown) time. The shadowed area is bound by the daily first and third quartile of the BFRA. Vertical dotted lines represent the time points in which the treatment condition changed, as displayed in the legend on top. Grey, dark red, and purple dots mark the representative days shown, respectively, in (**b**–**d**). (**b**) A representative day in cDBS+. Top left: daily evolution of the stimulation current for the left (black) and right (red) STN. Bottom left: daily evolution of the BFRA. Awake (8 a.m.–10 p.m.) and asleep (midnight to 6 a.m.) periods are separated by vertical solid lines. Bottom right: distribution of the BFRA separately for the awake (yellow) and asleep (brown) period. (**c**) Same as (**b**) for a representative day in aDBS+. (**d**) Same as (**b**) for a representative day in aDBS−. On these three days (**b**–**d**), the total electrical energy delivered (TEED) was comparable (values reported in W. Left STN, cDBS+: 1.2 × 10^−4^, aDBS+: 1.3 × 10^−4^, aDBS−: 1.3 × 10^−4^; right STN, cDBS+: 1.1 × 10^−4^, aDBS+: 1.1 × 10^−4^, aDBS−: 1.1 × 10^−4^). In aDBS mode, the TEED was calculated every minute and then averaged across the day. (**e**) Boxplot of the daily median BFRA during awake (yellow) and asleep (brown) time in cDBS+, aDBS+, and aDBS−. Top horizontal lines define significant differences (solid line: *p* < 0.001, significance set at 0.05). (**f**) Same as (**e**) for the interquartile range of the BFRA. Abbreviations: a, adaptive; BFRA, amplitude of the STN-LFP in the patient-specific beta frequency range; c, conventional; DBS, deep brain stimulation; DBS+, with dopaminergic medication; DBS−, without dopaminergic medication; LFP, local field potential and STN, subthalamic nucleus.

For this study, a total of 168 days in aDBS+, 47 days with cDBS+ and 63 days with aDBS− were collected. System usability and technical issues caused sporadic data loss (45 days with unavailable data over the 11-month follow-up). See legend in Figure 2a for the evolution in time of the stimulation condition.

During aDBS+ and cDBS+, dopaminergic therapy was stable and maintained (i.e., +) with levodopa/carbidopa 100/25 mg TID, opicapone 50 mg QD, and rasagiline 1 mg QD. The aDBS+ settings were: C+1−, 2.6–3.9 mA, 130 Hz, 80 µs (left STN); C+8−, 2.6–3.0 mA, 130 Hz, 80 µs (right STN) (see Methods, Figure 1 and Figure S1 [28,29,30]). The cDBS+ settings were: C+1−, 3.4 mA, 130 Hz, 80 µs (left STN), and C+8−, 2.8 mA, 130 Hz, 80 µs (right STN). The estimated total electrical energy delivered (TEED) was comparable between DBS modes (Figure 2b–d) [31].

The patient demonstrated significant and stable benefit from bilateral STN-cDBS+ as shown by Unified Parkinson Disease Rating Scale (UPDRS) parts III and IV scores of 17/108 and 6/108 with both Activa SC and AlphaDBS devices. The improvement was also remarkable with the AlphaDBS device in aDBS+ mode (UPDRS-III of 14/108 and UPDRS-IV of 3/108) and aDBS− mode (UPDRS-III of 8/108 and UPDRS-IV of 0/108, i.e., absence of dyskinesia). During brief discontinuation (about 30 min) of DBS treatment and after 12 h of withdrawal of dopaminergic medications, the UPDRS-III score was 57/108. The UPDRS scores refer to the last clinical assessments always performed in the late morning in stable chronic therapeutic conditions. The evaluating physician was blind to the treatment of the patient. Of note is that the patient preferred the aDBS stimulation mode over cDBS for better control of PD-related motor symptoms and greater ease and enjoyment in activities of daily living. He never complained of cognitive impairment, depression, anxiety or apathy, sleep problems, dysautonomia, hyposmia, or constipation. Genetic testing excluded common GBA, Park2, and PINK1 mutations. The patient is currently being followed up in aDBS for more than two years with remarkable and stable control of symptoms.

### 3.2. Long-Term Stable Beta-Band Peak Frequency

**Figure 3:**
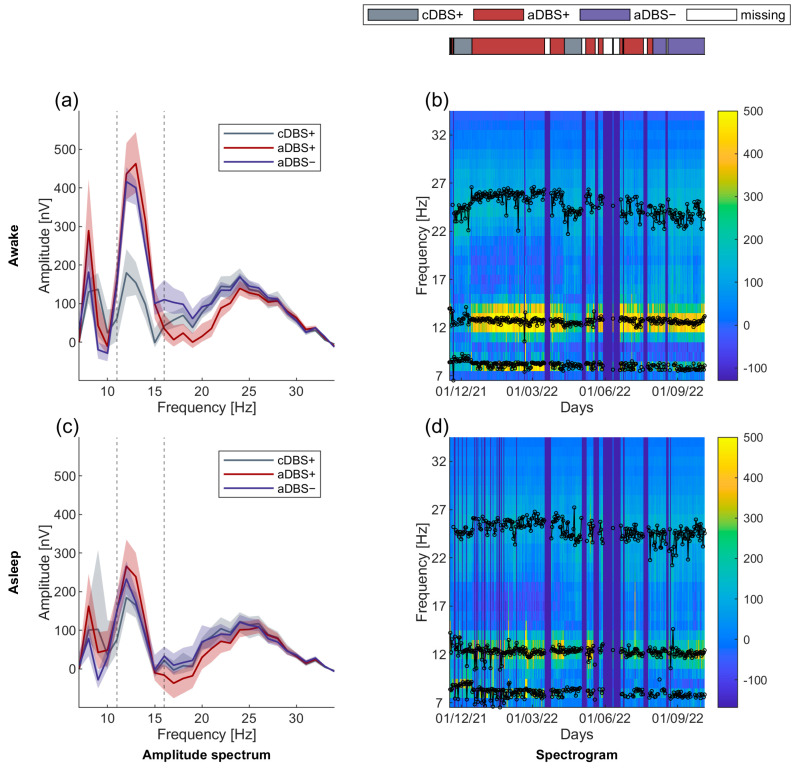
Long-term stable beta-band peak frequency. (**a**) Median (solid line) of the daily mean amplitude spectra throughout the 11 months of recording in the three treatment conditions (cDBS+ in grey, aDBS+ in dark red, and aDBS− in purple) during awake time. The dashed area is bound by the first and third quartiles of the daily mean amplitude spectra. (**b**) Spectrogram of daily mean amplitude spectra during awake time. Blue vertical lines correspond to missing or removed data periods due to residual spectral artifacts after neural power law component removal (see Section 2). Black lines on top of the spectrogram represent the time course of the central frequency of the three Gaussian peaks identified in each daily mean amplitude spectrum during awake time. (**c**) Same as (**a**) during asleep time. (**d**) Same as (**b**) during asleep time. Abbreviations: a, adaptive; c, conventional; DBS, deep brain stimulation; DBS+, with dopaminergic medication and DBS−, without dopaminergic medication.

In the three treatment conditions (cDBS+, aDBS+, and aDBS−), we acquired unilateral (left) STN-LFP spectrum every ten minutes, and we analyzed separately the spectra recorded during awake (8 a.m.–10 p.m.) and asleep (midnight to 6 a.m.) time (see Section 2). We identified three spectral peaks (see Section 2) in the 7–34 Hz range (values reported in Hz as median [first quartile, third quartile]: first peak: 8.2 [7.8, 8.3] (awake) and 8.2 [7.7, 8.4] (asleep); second peak within patient-specific beta range: 12.7 [12.5, 12.9] (awake) and 12.3 [12.1, 12.5] (asleep); third peak: 24.7 [23.9, 25.7] (awake) and 24.8 [24.3, 25.5] (asleep), Figure 3). Of note, the interquartile range of the beta peak was below 5% of the peak median value both during awake and asleep time, showing its stability across the whole recording period.

### 3.3. Larger Sleep-Wake Variation in Beta Amplitude in aDBS than in cDBS

From the spectra acquired every ten minutes, we computed the amplitude in the patient-specific beta frequency range (BFRA, see Section 2 and Figure 2). Then, for each day, we calculated the daily median and interquartile range of the BFRA separately for the awake and asleep periods (see Section 2).

We observed a statistically significant interaction between treatment (cDBS+, aDBS+, and aDBS−) and patient’s status (awake and asleep) in determining the daily median BFRA (values reported in nV as median [first quartile, third quartile]. Awake: cDBS+: 94.6 [61.4, 134.3], aDBS+: 220.3 [174, 279.5], aDBS−: 228.5 [189, 252.2]. Asleep: cDBS+: 94.5 [62, 110.6], aDBS+: 65.6 [5.6, 121.9], aDBS−: 80.7 [36.6, 135]. Mixed ANOVA on the ranks: F(2,296) = 21.23, *p* < 0.001, Figure 2e). Simple main effects analysis showed that treatment (*p* < 0.001) and patient’s status (*p* < 0.001) both had a statistically significant effect on the daily median BFRA. During awake time, this value was significantly higher in aDBS+ and aDBS− than in cDBS+ (Kruskal-Wallis: *p* < 0.001, Figure 2e and Table 1). During asleep, the daily median BFRA did not differ between treatments (Kruskal-Wallis: *p* = 0.15). A statistically significant difference in the daily median BFRA between awake and asleep time was observed for both the aDBS+ and aDBS− conditions (Wilcoxon signed-rank: *p* < 0.001) but not for cDBS+ (Figure 2e and Table 1).

### 3.4. Larger Variability in Beta Amplitude in aDBS than in cDBS

We found a significant interaction between treatment and patient’s status in determining the daily interquartile range of BFRA (values reported in nV as median [first quartile, third quartile]. Awake: cDBS+: 133.8 [114.9, 167.1], aDBS+: 612 [533.1, 690.3], aDBS−: 354 [287.8, 452.2]. Asleep: cDBS+: 144.6 [112, 161.3], aDBS+: 459.3 [339.8, 593.1], aDBS−: 390.5 [325.3, 488.4]. Mixed ANOVA on the ranks: F(2,296) = 16.73, *p* < 0.001, Figure 2f). Both factors had a statistically significant effect (*p* < 0.001). During awake time, the interquartile range was significantly higher in aDBS+ than in cDBS+ and aDBS−, and in aDBS− compared to cDBS+ (Kruskal-Wallis: *p* < 0.001, Figure 2f and Table 1). The same ranking was replicated during asleep time, but with no significant difference between aDBS+ and aDBS− (Kruskal-Wallis: *p* < 0.001, Figure 2f and Table 1). For the daily BFRA interquartile range, we also observed a reduction in asleep compared to awake time but in aDBS+ only (Wilcoxon signed-rank: *p* < 0.001, Figure 2f and Table 1).

### 3.5. STN-LFP Beta Amplitude in aDBS Was Informative about Awake and Asleep State

In both aDBS+ and aDBS−, the median BFRA contained significant information about the awake and asleep condition (0.32 and 0.42 bits, respectively, *p* < 0.05, see Section 2), but its interquartile range only carried significant information in aDBS+ (0.20 bits, *p* < 0.05 in aDBS+ vs. 0.02 bits, *p* > 0.1 in aDBS−). Neither feature carried information about the awake and asleep condition in cDBS+ (0.01 bits, *p* > 0.1).

## 4. Discussion

The aDBS paradigm described, with linear current modulation, proved to be effective in the long-term control of Parkinsonian motor symptoms and improvement of patient well-being. From a pathophysiological point of view, our data showed overall stability of the STN beta-frequency peaks over time and a different modulation of the beta amplitude and fluctuations with aDBS compared with cDBS.

The subjective (i.e., reported by the patient) and objective superior clinical benefit of aDBS over cDBS, and the gradual discontinuation of all pharmacological treatment, should be considered as a novel condition—possibly a functional recovery—permitted by a putative (re)activation of compensatory basal ganglia–thalamic–cortical circuitries yet to be identified. A brain–computer interface, driven by algorithms for stimulation delivery and adaptation, could result not only in an immediate benefit on the Parkinsonian symptoms but also in a more significant long-term comprehensive improvement, possibly permitted by the restoration of more physiological neural activities (e.g., beta oscillations). This perspective is reflected in our observations, which show greater beta oscillatory activity in aDBS than in cDBS, paralleling the clinical benefit over time. While cDBS would exclusively retain a suppressive action on pathological neural activity, aDBS may permit and/or promote a more profound integration of the functional and informative [32,33] components of the beta oscillations while retaining the positive effect on Parkinsonian symptoms. In line with this hypothesis, we think that the reduced subthalamic beta activity during sleep depends on the lower involvement of the STN compared with voluntary motor control in the nocturnal regulation of sleep-related rhythmic and homeostatic processes. This reasoning may also justify the different responses to chronic (over months) or acute (during a pharmacological test with levodopa) aDBS [11,34]. In the latter case, only the suppressive response on akinetic-rigid signs would be evident. The significant information carried by STN beta amplitude on the sleep-wake cycle further promises more informed programming of adaptive DBS stimulation and improved aDBS algorithms, also considering the influence of the individual circadian rhythms.

Our recordings also demonstrate the overall stability of beta-band peaks over time. This result is important for current aDBS algorithms because it testifies to the capability of correctly monitoring power modulations over an established range of frequencies. However, we must acknowledge that we were unable to correlate subthalamic activity and kinematic parameters to allow the description of specific changes in neural activity (e.g., frequency modulation [33]) under certain conditions, such as walking. Indeed, there is increasing evidence that the beta peak frequency is an important and functionally relevant parameter of oscillatory activity both at a cortical [35] and subcortical [33] level.

To conclude, we reported subthalamic recordings and clinical monitoring of a PD patient treated for almost one year with aDBS alternated with short periods of cDBS. We observed higher and more variable beta amplitude of STN-LFPs, in parallel with clinical benefit, in aDBS compared to cDBS. Despite reporting results from one single patient, our clinical case provides the preliminary first evidence of the clinical efficacy of aDBS over one year, paving the way for new studies with more reliable neuromodulation strategies based on a continuous bidirectional brain–computer communication—not only to better tailor symptomatic improvement but also to possibly (re)activate compensatory brain resources.

## Figures and Tables

**Table 1:** Statistical comparisons in the patient-specific beta range. Comparison of the daily median and interquartile range of the BFRA between the three different treatment conditions (cDBS+, aDBS+ and aDBS−) and the two different patient’s states (awake and asleep). The significance level was set to 0.05. Effect size was calculated with rCoh (see Section 2) and reported for significant comparisons. Abbreviations: a, adaptive; BFRA, patient-specific beta frequency range amplitude; c, conventional; DBS, deep brain stimulation; DBS+, with dopaminergic medication; DBS−, without dopaminergic medication and rCoh, Robust Cohen’s distance.

	Factors			Median F(2,296) = 21.23, *p* < 0.001		Interquartile Range F(2,296) = 16.73, *p* < 0.001	
	Factor 1	Factor 2	Factor 3	*p*-Value	rCoh	*p*-Value	rCoh
Awake	cDBS+	aDBS+	aDBS−	<0.001		<0.001	
	cDBS+	aDBS+		<0.001	−1.69	<0.001	−4.04
	cDBS+	aDBS−		<0.001	−2.32	<0.001	−2.39
	aDBS+	aDBS−		1		<0.001	1.95
Asleep	cDBS+	aDBS+	aDBS−	0.15		<0.001	
	cDBS+	aDBS+				<0.001	−1.86
	cDBS+	aDBS−				<0.001	−2.68
	aDBS+	aDBS−				0.19	
cDBS+	Awake	Asleep		0.058		0.92	
aDBS+	Awake	Asleep		<0.001	1.86	<0.001	0.91
aDBS−	Awake	Asleep		<0.001	2.10	0.17	

## Data Availability

LFPs recorded with the AlphaDBS device cannot be deposited in a public repository because they can be traceable to the identity of the subject. They will be made available upon reasonable request to the lead contact. No original method has been developed. All analyses were performed in MATLAB 2021a and MATLAB 2023a (The MathWorks Inc., Natik, MA, USA) with standard functions. Any additional information required to reanalyze the data reported in this paper is available from the lead contact upon request.

## Supplementary Materials

The following supporting information can be downloaded at: https://www.mdpi.com/article/10.3390/bioengineering11100990/s1, Figure S1: DBS electrode positioning. (a) 2D localization of the stimulating or recording contacts (white open circles, for right and left STN). The motor subregion of the STN is shown in orange. Contacts enumeration of the quadripolar electrodes follows this rule: 0 or 8 are the ventralmost contacts, 3 or 11 are the dorsalmost contacts, respectively for the left and right STN. Contacts 1 (left STN) and 8 (right STN) were chosen for stimulation as the ones giving best clinical outcome, while contact pair 0-2 in the left STN was chosen for sensing as the one showing the most prominent beta peak. (b) 3D localization of the stimulating or recording contacts (for right and left STN). The volume of tissue activated (VTA) is indicated in red color, while the subthalamic subregions are shown as follows: orange, sensorimotor STN, yellow, limbic STN, and light blue, associative STN. First and second row corresponds to VTA calculated with Amin (i.e., 2.6 mA for both left and right STN) and Amax (i.e., 3.9 mA for left STN and 3.0 mA for right STN), respectively. The stimulation frequency (130 Hz) and pulse width (80 µs) remain fixed bilaterally. (c) Left: demonstration that the stimulating contacts are very close to or adjacent to correlated sweet spots for best motor improvement. Beta peak is indicated in golden orange. Right: both right and left VTAs involve the side-specific sweet spots. DBS electrodes and contacts were localized based on pre- and postoperative neuroimaging using a tool designed for this task (as implemented in Lead-DBS software [1]). Atlas used for 2D and 3D visualization: Electrophysiological Atlas of STN Activity [2]. Sweet spots were calculated and visualized according to [3]. Abbreviations: A; predefined, clinically effective amplitude; DBS, deep brain stimulation, GPi, globus pallidus internus, RN, red nucleus, STN, subthalamic nucleus and VTA, volume of activated tissue.

## References

[1] Deuschl G., Schade-Brittinger C., Krack P., Volkmann J., Schäfer H., Bötzel K., Daniels C., Deutschländer A., Dillmann U., Eisner W. (2006). A Randomized Trial of Deep-Brain Stimulation for Parkinson’s Disease. N. Engl. J. Med..

[2] Pozzi N.G., Isaias I.U., Quartarone A., Ghilardi M.F., Boller F. (2022). Chapter 19—Adaptive Deep Brain Stimulation: Retuning Parkinson’s Disease. Handbook of Clinical Neurology.

[3] Chen C.C., Brücke C., Kempf F., Kupsch A., Lu C.S., Lee S.T., Tisch S., Limousin P., Hariz M., Brown P. (2006). Deep Brain Stimulation of the Subthalamic Nucleus: A Two-Edged Sword. Curr. Biol..

[4] Rodriguez-Oroz M.C., Moro E., Krack P. (2012). Long-Term Outcomes of Surgical Therapies for Parkinson’s Disease. Mov. Disord..

[5] St George R.J., Nutt J.G., Burchiel K.J., Horak F.B. (2010). A Meta-Regression of the Long-Term Effects of Deep Brain Stimulation on Balance and Gait in PD. Neurology.

[6] Reich M.M., Brumberg J., Pozzi N.G., Marotta G., Roothans J., Åström M., Musacchio T., Lopiano L., Lanotte M., Lehrke R. (2016). Progressive Gait Ataxia Following Deep Brain Stimulation for Essential Tremor: Adverse Effect or Lack of Efficacy?. Brain.

[7] Guidetti M., Marceglia S., Loh A., Harmsen I.E., Meoni S., Foffani G., Lozano A.M., Moro E., Volkmann J., Priori A. (2021). Clinical Perspectives of Adaptive Deep Brain Stimulation. Brain Stimul..

[8] Neumann W.-J., Degen K., Schneider G.-H., Brücke C., Huebl J., Brown P., Kühn A.A. (2016). Subthalamic Synchronized Oscillatory Activity Correlates With Motor Impairment in Patients With Parkinson’s Disease. Mov. Disord..

[9] Kühn A.A., Kupsch A., Schneider G.-H., Brown P. (2006). Reduction in Subthalamic 8-35 Hz Oscillatory Activity Correlates with Clinical Improvement in Parkinson’s Disease. Eur. J. Neurosci..

[10] Kühn A.A., Kempf F., Brücke C., Doyle L.G., Martinez-Torres I., Pogosyan A., Trottenberg T., Kupsch A., Schneider G.-H., Hariz M.I. (2008). High-Frequency Stimulation of the Subthalamic Nucleus Suppresses Oscillatory β Activity in Patients with Parkinson’s Disease in Parallel with Improvement in Motor Performance. J. Neurosci..

[11] Bocci T., Prenassi M., Arlotti M., Cogiamanian F.M., Borellini L., Moro E., Lozano A.M., Volkmann J., Barbieri S., Priori A. (2021). Eight-Hours Conventional versus Adaptive Deep Brain Stimulation of the Subthalamic Nucleus in Parkinson’s Disease. npj Parkinson’s Dis..

[12] Arlotti M., Marceglia S., Foffani G., Volkmann J., Lozano A.M., Moro E., Cogiamanian F., Prenassi M., Bocci T., Cortese F. (2018). Eight-Hours Adaptive Deep Brain Stimulation in Patients with Parkinson Disease. Neurology.

[13] Little S., Pogosyan A., Neal S., Zavala B., Zrinzo L., Hariz M., Foltynie T., Limousin P., Ashkan K., FitzGerald J. (2013). Adaptive Deep Brain Stimulation in Advanced Parkinson Disease. Ann. Neurol..

[14] Little S., Beudel M., Zrinzo L., Foltynie T., Limousin P., Hariz M., Neal S., Cheeran B., Cagnan H., Gratwicke J. (2016). Bilateral Adaptive Deep Brain Stimulation Is Effective in Parkinson’s Disease. J. Neurol. Neurosurg. Psychiatry.

[15] Rosa M., Arlotti M., Ardolino G., Cogiamanian F., Marceglia S., Di Fonzo A., Cortese F., Rampini P.M., Priori A. (2015). Adaptive Deep Brain Stimulation in a Freely Moving Parkinsonian Patient. Mov. Disord..

[16] Rosa M., Arlotti M., Marceglia S., Cogiamanian F., Ardolino G., Fonzo A.D., Lopiano L., Scelzo E., Merola A., Locatelli M. (2017). Adaptive Deep Brain Stimulation Controls Levodopa-Induced Side Effects in Parkinsonian Patients: DBS Controls Levodopa-Induced Side Effects. Mov. Disord..

[17] Little S., Tripoliti E., Beudel M., Pogosyan A., Cagnan H., Herz D., Bestmann S., Aziz T., Cheeran B., Zrinzo L. (2016). Adaptive Deep Brain Stimulation for Parkinson’s Disease Demonstrates Reduced Speech Side Effects Compared to Conventional Stimulation in the Acute Setting. J. Neurol. Neurosurg. Psychiatry.

[18] Isaias I.U., Benti R., Cilia R., Canesi M., Marotta G., Gerundini P., Pezzoli G., Antonini A. (2007). [123I]FP-CIT Striatal Binding in Early Parkinson’s Disease Patients with Tremor vs. Akinetic-Rigid Onset. Neuroreport.

[19] Arlotti M., Colombo M., Bonfanti A., Mandat T., Lanotte M.M., Pirola E., Borellini L., Rampini P., Eleopra R., Rinaldo S. (2021). A New Implantable Closed-Loop Clinical Neural Interface: First Application in Parkinson’s Disease. Front. Neurosci..

[20] Deep Brain Stimulation Programming: Mechanisms, Principles and Practice-Montgomery Jr, Erwin B: 9780190259600—AbeBooks. https://www.abebooks.it/9780190259600/Deep-Brain-Stimulation-Programming-Mechanisms-0190259604/plp.

[21] Değirmenci Y. (2024). Current DBS Programming. Deep Brain Stimul..

[22] van Rheede J.J., Feldmann L.K., Busch J.L., Fleming J.E., Mathiopoulou V., Denison T., Sharott A., Kühn A.A. (2022). Diurnal Modulation of Subthalamic Beta Oscillatory Power in Parkinson’s Disease Patients during Deep Brain Stimulation. npj Parkinson’s Dis..

[23] Donoghue T., Haller M., Peterson E.J., Varma P., Sebastian P., Gao R., Noto T., Lara A.H., Wallis J.D., Knight R.T. (2020). Parameterizing Neural Power Spectra into Periodic and Aperiodic Components. Nat. Neurosci..

[24] Johnson M. Mixed (Between/Within Subjects) ANOVA. https://it.mathworks.com/matlabcentral/fileexchange/27080-mixed-between-within-subjects-anova.

[25] Algina J., Keselman H.J., Penfield R.D. (2005). An Alternative to Cohen’s Standardized Mean Difference Effect Size: A Robust Parameter and Confidence Interval in the Two Independent Groups Case. Psychol. Methods.

[26] Panzeri S., Treves A. (1996). Analytical Estimates of Limited Sampling Biases in Different Information Measures. Network.

[27] Magri C., Whittingstall K., Singh V., Logothetis N.K., Panzeri S. (2009). A Toolbox for the Fast Information Analysis of Multiple-Site LFP, EEG and Spike Train Recordings. BMC Neurosci..

[28] Horn A., Kühn A.A. (2015). Lead-DBS: A Toolbox for Deep Brain Stimulation Electrode Localizations and Visualizations. NeuroImage.

[29] Horn A., Neumann W.-J., Degen K., Schneider G.-H., Kühn A.A. (2017). Toward an Electrophysiological “Sweet Spot” for Deep Brain Stimulation in the Subthalamic Nucleus. Hum. Brain Mapp..

[30] Horn A., Kühn A.A., Merkl A., Shih L., Alterman R., Fox M. (2017). Probabilistic Conversion of Neurosurgical DBS Electrode Coordinates into MNI Space. NeuroImage.

[31] Wilkins K.B., Petrucci M.N., Lambert E.F., Melbourne J.A., Gala A.S., Akella P., Parisi L., Cui C., Kehnemouyi Y.M., Hoffman S.L. (2024). Beta Burst-Driven Adaptive Deep Brain Stimulation Improves Gait Impairment and Freezing of Gait in Parkinson’s Disease. arXiv.

[32] Vissani M., Palmisano C., Volkmann J., Pezzoli G., Micera S., Isaias I.U., Mazzoni A. (2021). Impaired Reach-to-Grasp Kinematics in Parkinsonian Patients Relates to Dopamine-Dependent, Subthalamic Beta Bursts. npj Parkinson’s Dis..

[33] Canessa A., Palmisano C., Isaias I.U., Mazzoni A. (2020). Gait-Related Frequency Modulation of Beta Oscillatory Activity in the Subthalamic Nucleus of Parkinsonian Patients. Brain Stimul..

[34] Arlotti M., Palmisano C., Minafra B., Todisco M., Pacchetti C., Canessa A., Pozzi N.G., Cilia R., Prenassi M., Marceglia S. (2019). Monitoring Subthalamic Oscillations for 24 Hours in a Freely Moving Parkinson’s Disease Patient. Mov. Disord..

[35] Kilavik B.E., Ponce-Alvarez A., Trachel R., Confais J., Takerkart S., Riehle A. (2012). Context-Related Frequency Modulations of Macaque Motor Cortical LFP Beta Oscillations. Cerebral. Cortex..

