# Supplementary material for: Adaptive vs. Conventional Deep Brain Stimulation: One-Year Subthalamic Recordings and Clinical Monitoring in a Patient with Parkinson’s Disease
Source: Bioengineering (Basel). 2024 Sep 30;11(10):990. doi: 10.3390/bioengineering11100990 (PMC11504236; doi:10.3390/bioengineering11100990)
Supplement: Supplementary file 1 [file bioengineering-11-00990-s001.zip › bioengineering-3164948-supplementary.pdf]

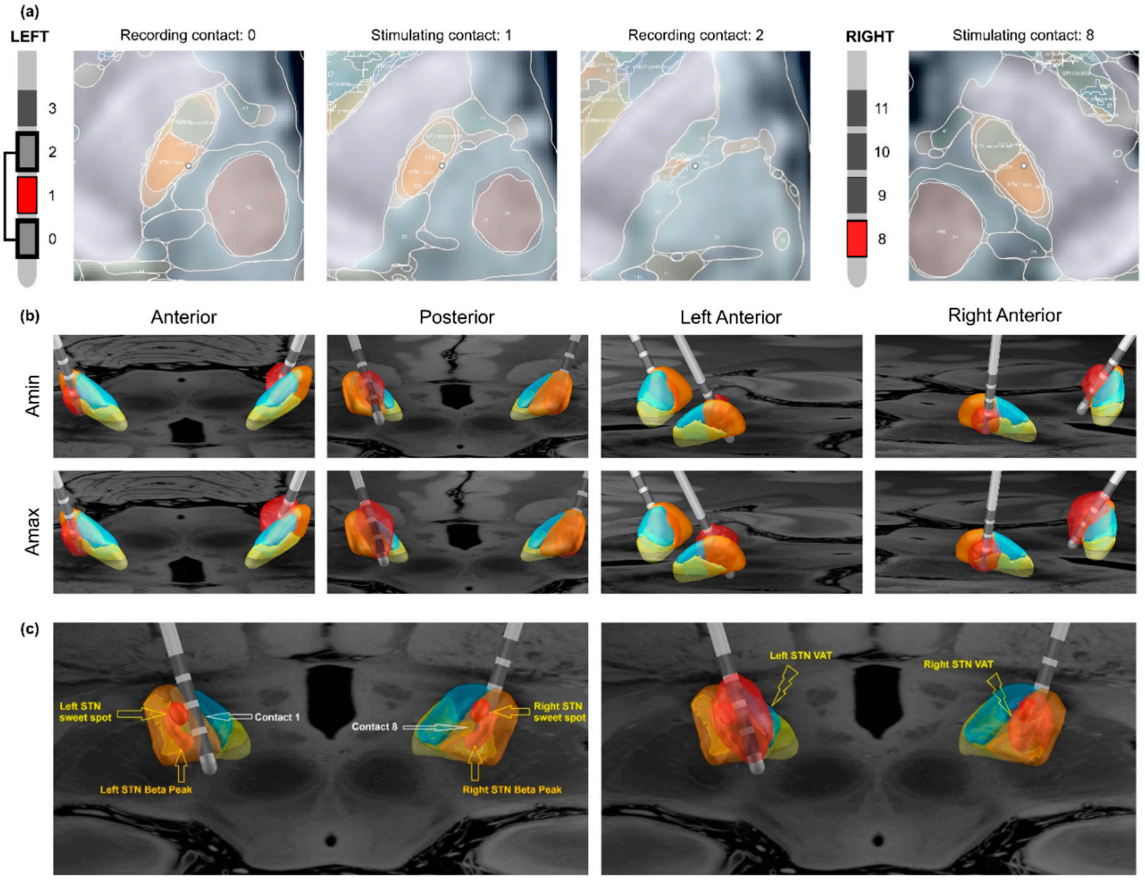

Figure S1: DBS electrode positioning. (a) 2D localization of the stimulating or recording contacts (white open circles, for right and left STN). The motor subregion of the STN is shown in orange. Contacts enumeration of the quadripolar electrodes follows this rule: 0 or 8 are the ventralmost contacts, 3 or 11 are the dorsalmost contacts, respectively for the left and right STN. Contacts 1 (left STN) and 8 (right STN) were chosen for stimulation as the ones giving best clinical outcome, while contact pair 0-2 in the left STN was chosen for sensing as the one showing the most prominent beta peak. (b) 3D localization of the stimulating or recording contacts (for right and left STN). The volume of tissue activated (VTA) is indicated in red color, while the subthalamic subregions are shown as follows: orange, sensorimotor STN, yellow, limbic STN, and light blue, associative STN. First and second row corresponds to VTA calculated with Amin (i.e., 2.6 mA for both left and right STN) and Amax (i.e., 3.9 mA for left STN and 3.0 mA for right STN), respectively. The stimulation frequency (130 Hz) and pulse width (80 µs) remain fixed bilaterally. (c) Left: demonstration that the stimulating contacts are very close to or adjacent to correlated sweet spots for best motor improvement. Beta peak is indicated in golden orange. Right: both right and left VTAs involve the side-specific sweet spots. DBS electrodes and contacts were localized based on pre- and postoperative neuroimaging using a tool designed for this task (as implemented in Lead-DBS software [28]). Atlas used for 2D and 3D visualization: Electrophysiological Atlas of STN Activity [29]. Sweet spots were calculated and visualized according to [30]. Abbreviations: A; predefined, clinically effective amplitude; DBS, deep brain stimulation, GPi, globus pallidus internus, RN, red nucleus, STN, subthalamic nucleus and VTA, volume of activated tissue.

BIBLIOGRAPHY

28. Horn, A.; Kühn, A.A. Lead-DBS: A Toolbox for Deep Brain Stimulation Electrode Localizations and Visualizations. *NeuroImage* **2015**, *107*, 127–135, doi:10.1016/j.neuroimage.2014.12.002.
29. Horn, A.; Neumann, W.-J.; Degen, K.; Schneider, G.-H.; Kühn, A.A. Toward an Electrophysiological "Sweet Spot" for Deep Brain Stimulation in the Subthalamic Nucleus. *Hum Brain Mapp* **2017**, *38*, 3377–3390, doi:10.1002/hbm.23594.
30. Horn, A.; Kühn, A.A.; Merkl, A.; Shih, L.; Alterman, R.; Fox, M. Probabilistic Conversion of Neurosurgical DBS Electrode Coordinates into MNI Space. *NeuroImage* **2017**, *150*, 395–404, doi:10.1016/j.neuroimage.2017.02.004.
